# Neural, physiological, and behavioral correlates of visuomotor cognitive load

**DOI:** 10.1038/s41598-017-07897-z

**Published:** 2017-08-18

**Authors:** S. M. Hadi Hosseini, Jennifer L. Bruno, Joseph M. Baker, Andrew Gundran, Lene K. Harbott, J. Christian Gerdes, Allan L. Reiss

**Affiliations:** 1 Center for Interdisciplinary Brain Sciences Research, Department of Psychiatry and Behavioral Sciences, Stanford University, 401 Quarry Road, Stanford, CA 94305-5795 USA; 2 Department of Mechanical Engineering, Stanford University, 473 Oak Road, Stanford, CA 94305 USA; 3 Departments of Radiology and Pediatrics, Stanford University, 401 Quarry Road, Stanford, CA 94305 USA

## Abstract

Visuomotor ability is quite crucial for everyday functioning, particularly in driving and sports. While there is accumulating evidence regarding neural correlates of visuomotor transformation, less is known about the brain regions that accommodate visuomotor mapping under different cognitive demands. We concurrently measured cortical activity and pupillary response, using functional near infrared spectroscopy (fNIRS) and eye-tracking glasses, to examine the neural systems linked to pupil dilation under varying cognitive demands. Twenty-three healthy adults performed two sessions of a navigation task, in which the cognitive load was manipulated by either reversing the visuomotor mapping or increasing the speed of the moving object. We identified a region in the right superior parietal lobule that responded to both types of visuomotor load and its activity was associated with larger pupillary response and better performance in the task. Our multimodal analyses suggest that activity in this region arises from the need for increased attentional effort and alertness for visuomotor control and is an ideal candidate for objective measurement of visuomotor cognitive load. Our data extend previous findings connecting changes in pupil diameter to neural activity under varying cognitive demand and have important implications for examining brain-behavior associations in real-world tasks such as driving and sports.

## Introduction

Visuomotor ability is crucial for everyday functioning, particularly in driving and sports. Functional neuroimaging evidence indicates the involvement of various brain regions in visuomotor transformation processes including the parietal, prefrontal and premotor cortices as well as subcortical structures including the striatal and cerebellar network^1–6^. Specifically, parietal and prefrontal cortices play an important role in remapping visuomotor associations^5, 7, 8^ before the learned mapping is consolidated in the striatal and cerebellar networks^6, 9, 10^. The parietal cortex – specifically the superior parietal lobule – acts as a multifaceted behavioral integrator that binds visuospatial, motor, and cognitive information into a topographically organized signal of behavioral salience^3^ while the medial prefrontal and anterior cingulate regions are primarily engaged by the need to select among competing response alternatives^11^. In addition, adaptation to a novel visuomotor mapping engages attentional and visuospatial working memory processes subserved by the inferior parietal lobule and the dorsolateral prefrontal cortex^8^.

**Figure 1:**
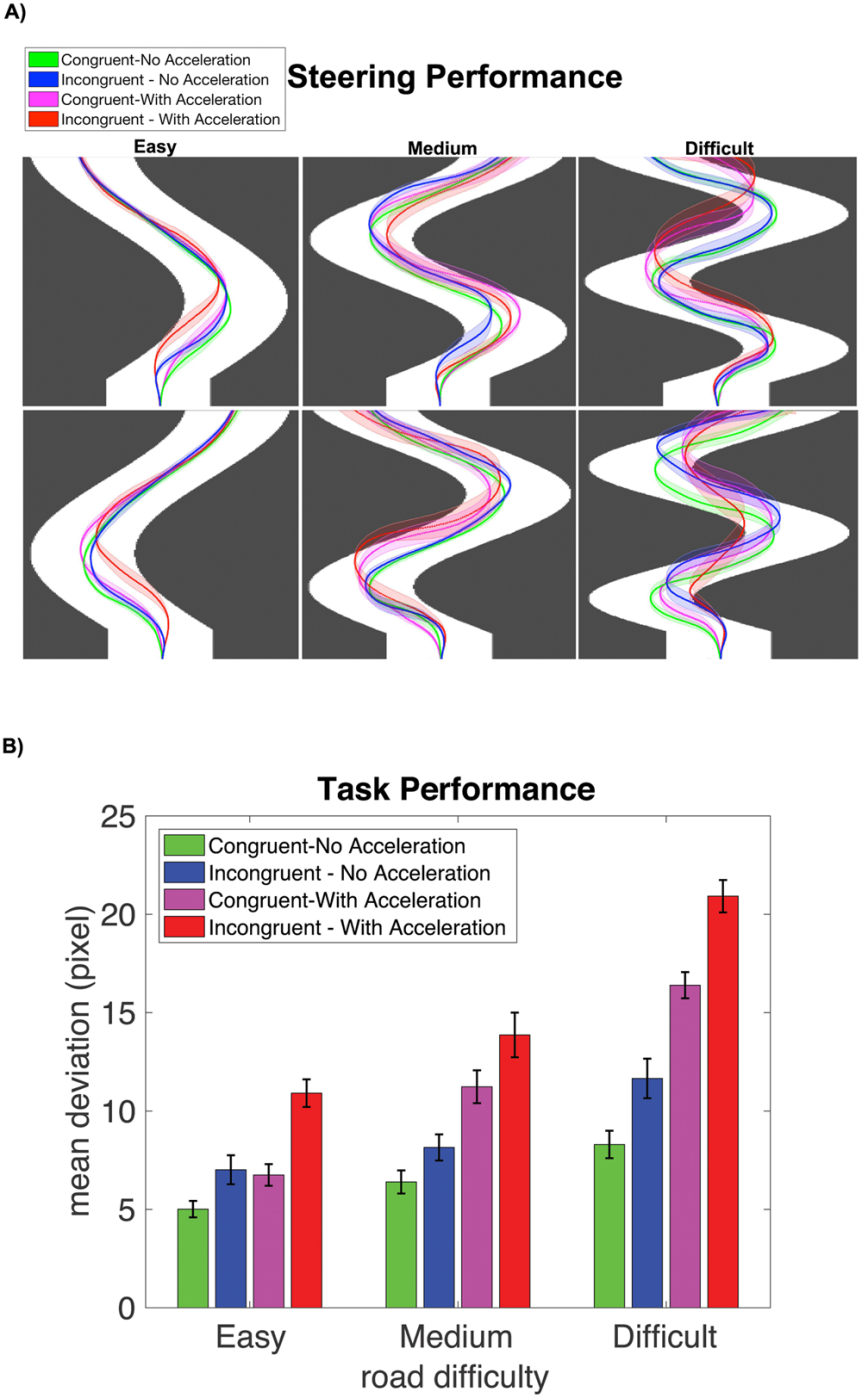
Average steering performance across conditions. (**A**) The shaded lines show mean (standard error) of the steering path across different conditions. The green (pink) lines indicate congruent steering with (w/o) acceleration and blue (red) lines indicate incongruent steering with (w/o) acceleration. (**B**) Mean (standard error) of deviation from the center of the road across different conditions. The green (pink) bars indicate congruent steering with (w/o) acceleration and blue (red) bars indicate incongruent steering with (w/o) acceleration.

While several studies have examined the neural systems underlying visuomotor control in humans, less is known about the regions that accommodate visuomotor mapping under different cognitive demands. In this study, we employed functional near infrared spectroscopy (fNIRS) to examine the neural correlates of visuomotor mapping under varying cognitive loads. Specifically, we designed a computer-based visuomotor task, in which the participant was asked to keep a moving dot in the middle of a path (Fig. 1A) using the left and right keyboard arrows. We manipulated the cognitive load by reversing the mapping between visual and motor response (right/left arrow moved the dot to the left/right) and also by increasing the speed of the moving dot. We aimed to identify the distinct and overlapping brain regions that respond to increased visuomotor cognitive load associated with reverse visuomotor mapping and increased speed.

Accumulating evidence suggests that pupil size varies in response to cognitive load and attentional demands of the task^12–15^. Specifically, pupillary change in response to cognitive processing is linked to activity in the locus coeruleus noradrenergic system which is thought to modulate the attentional network^13, 16^. In a simultaneous fMRI and pupilometry study on a multiple object tracking task, Alnas and colleagues (2014) found pupil-related activity in the locus coeruleus and a number of other structures including the dorsal attention network, supporting the link between pupil dilations and cognitive workload. We concurrently measured fNIRS and pupil dilation for the first time to examine the neural systems linked to pupil dilation that respond to visuomotor cognitive load.

Several studies investigated the dynamics of changes in brain activity in the course of adaptation to visuomotor transformation^8, 9, 17–22^. While these studies indicated the involvement of prefrontal regions in early stage of learning novel visuomotor mapping^8, 9, 17^, evidence regarding the involvement of parietal regions in adaptation to novel visuomotor mapping is controversial, signaling the involvement of these regions in both early and late stages of learning^8, 18–22^. We separately examined the profile of brain activity and pupil response in early and later stages of the visuomotor task to distinguish the brain regions adapting to novel visuomotor mapping and increased speed. We also explored the cortical regions that adapt to the visuomotor task on a trial-by-trial basis.

To our knowledge, this is the first study that examines neural and pupillary responses to different types of visuomotor cognitive loads using fNIRS. We employed wearable NIRS and eye tracking systems so that we could compare our current findings in a laboratory setting to real-world scenarios that involve visuomotor mapping of varying cognitive load, such as in driving and playing sports.

## Results

### Behavioral results

A repeated measures ANOVA was conducted to compare the main effect of steering (congruent and incongruent), acceleration (no acceleration, with acceleration) and road difficulty (easy, medium, hard) and their interactions on average deviation from the center of the road. We found a significant main effect of steering (F_1,19_ = 45.1, p < 0.0001), acceleration (F_1,19_ = 188.4, p < 0.0001) and road difficulty (F_2,18_ = 119.7, p < 0.0001) indicating poorer performance in incongruent and acceleration trials and in trials with difficult roads (Fig. 1B). The interaction of steering and acceleration (F_1,19_ = 4.6, p = 0.046), steering and road difficulty (F_2,18_ = 5.5, p = 0.009), and acceleration and road difficulty (F_2,18_ = 14.9, p < 0.001) were also significant suggesting that acceleration and incongruent steering result in poorer performance when combined either together or with more difficult roads. The three-way interaction among steering, acceleration and road difficulty was not significant (F_2,18_ = 0.55, p = 0.47).

Also, we separately examined the performance in early and later stages of the visuomotor task. In the first session of the task, we found a significant main effect of steering (F_1,19_ = 93.7, p < 0.0001) and acceleration (F_1,19_ = 102.8, p < 0.0001) indicating poorer performance (larger deviation) in incongruent and acceleration trials. We also found a significant steering by acceleration interaction (F_1,19_ = 44.9, p < 0.0001) indicating that acceleration results in poorer performance when combined with incongruent steering. In the second session, the main effect of steering did not remain significant (F_1,19_ = 1.93, p = 0.18) but we found a significant main effect of acceleration (F_1,19_ = 129.5, p < 0.0001) and a significant interaction effect (F_1,19_ = 5.15, p = 0.035).

### Pupillary response results

**Figure 2:**
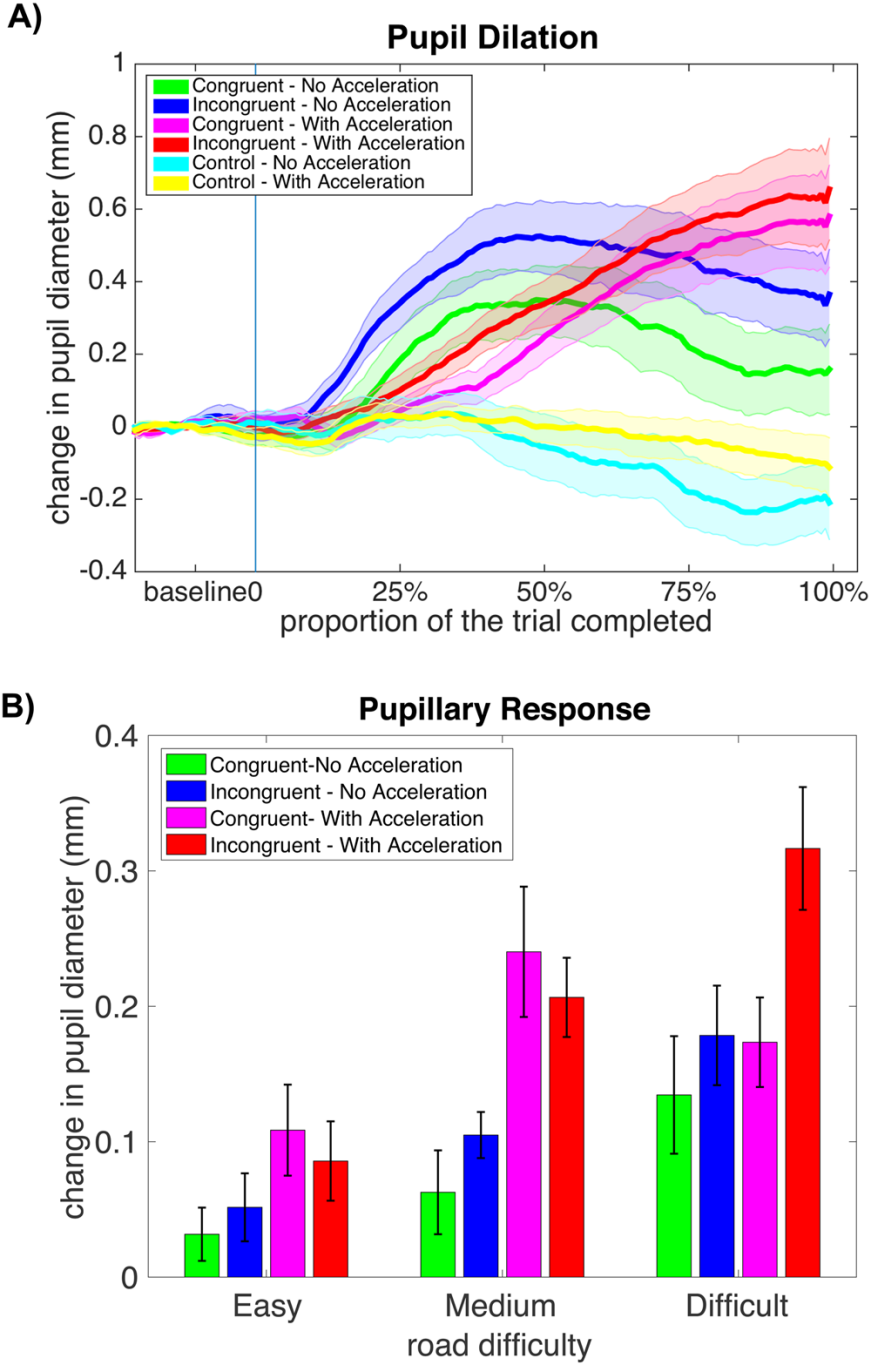
Pupillary responses. (**A**) The shaded lines show mean (standard error) of the changes in pupil diameter (relative to baseline) over the course of the trial across different conditions. Green (pink) lines indicate congruent steering with (w/o) acceleration, blue (red) lines indicate incongruent steering with (w/o) acceleration, and cyan (yellow) lines indicate control trials with (w/o) acceleration. (**B**) Mean (standard error) of changes in pupil diameter across different conditions. The green (pink) bars indicate congruent steering with (w/o) acceleration and blue (red) bars indicate incongruent steering with (w/o) acceleration.

The repeated measures ANOVA on pupil dilation revealed a significant main effect of steering (F_1,11_ = 11.03, p = 0.007), acceleration (F_1,11_ = 21.2, p = 0.001) and road difficulty (F_2,10_ = 20.1, p < 0.001) indicating larger pupil dilation in incongruent and acceleration trials and in trials with difficult roads (Fig. 2). We also found a significant steering by road interaction indicating larger pupil diameter in incongruent trials with difficult road type (F_2,10_ = 13.8, p < 0.001).

Further, we separately examined the pupil dilation in early and later stages of the visuomotor task. Consistent with behavioral results, the ANOVA for pupillary data showed significant main effects of steering (F_1,11_ = 11.0, p = 0.007) and acceleration (F_1,11_ = 31.2, p = 0.0002) in the first session, indicating larger pupil dilation in incongruent and acceleration trials. The steering by acceleration interaction was not significant (F_1,11_ = 0.10, p = 0.76). In the second session, we did not observe any significant effects (p’s > 0.11).

### Cortical activation results

**Figure 3:**
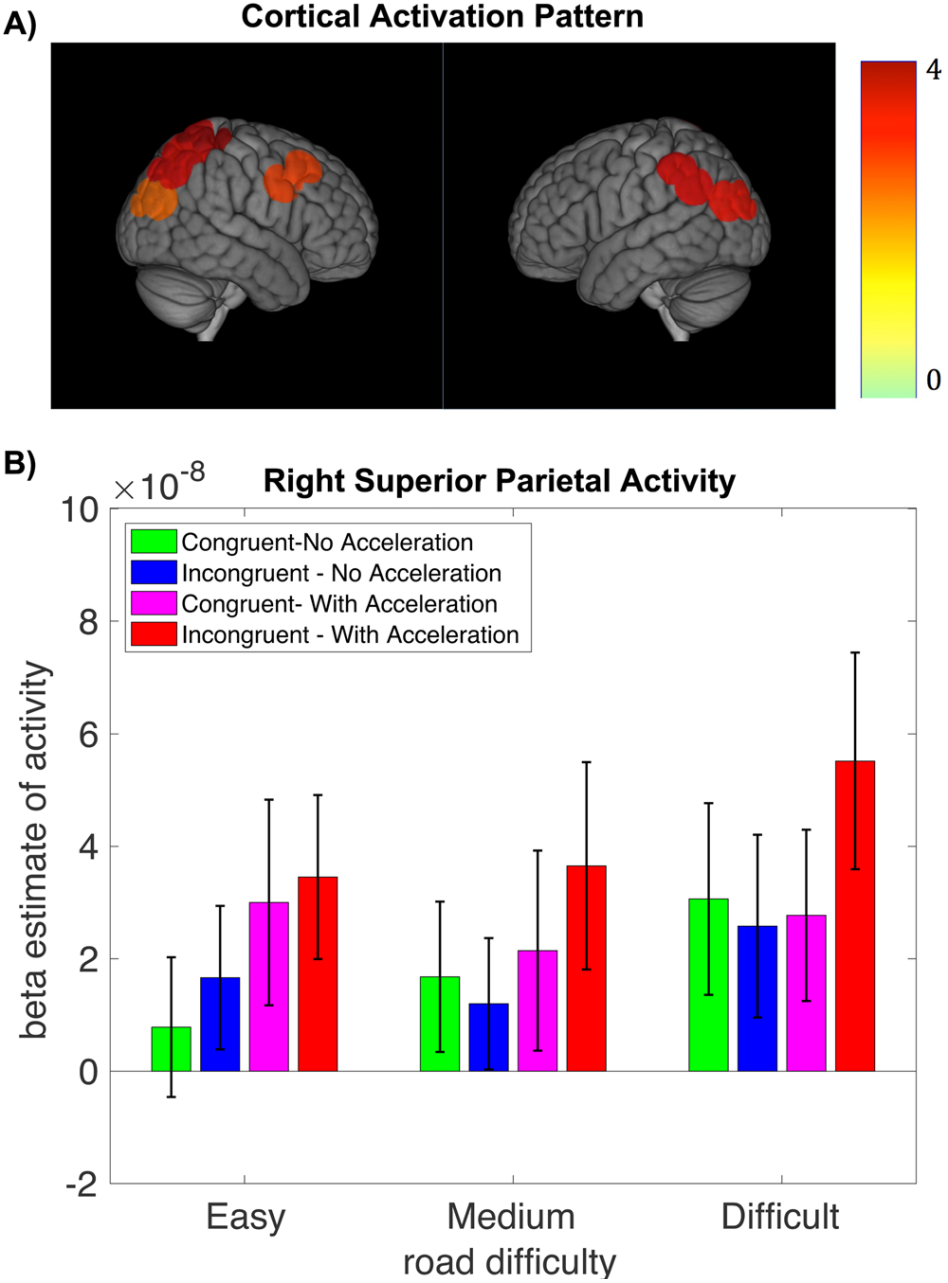
Profile of cortical brain activity. (**A**) The cortical regions with significantly greater activity in task compared with control (p < 0.05, FDR corrected). (**B**) The mean (standard error) of beta estimate of activity in the right superior parietal region across conditions. The green (pink) bars indicate congruent steering with (w/o) acceleration and blue (red) bars indicate incongruent steering with (w/o) acceleration.

The cortical regions that showed significantly greater activity in the task compared with control condition included the right middle frontal gyrus, right superior parietal and bilateral inferior parietal lobules (p < 0.05, FDR corrected) (Fig. 3). The repeated measures ANOVA on beta estimates of activity in the task conditions revealed a significant main effect of acceleration in the left inferior and right superior parietal regions (p < 0.05, FDR corrected). Also, we separately examined the profile of brain activity in early and later stages of the visuomotor task to distinguish the brain regions adapting to novel visuomotor mapping and increased speed. The repeated measures ANOVA revealed a significant main effect of steering, main effect of acceleration, and a significant interaction between steering and acceleration in the first session (p < 0.05, FDR corrected). This analysis indicated greater activity in the right superior parietal lobule in response to incongruent and acceleration trials. The activity in this region also showed a significant steering by acceleration interaction indicating greater activity in acceleration trials when combined with incongruent steering. On the other hand, activity in bilateral inferior parietal lobule only showed significant increase in response to acceleration. In the second session, we only found a significant main effect of steering indicating greater activity in the right superior parietal region in response to incongruent steering trials (p < 0.05, FDR corrected).

Further, using a parametric model, we explored the cortical regions that adapt to the visuomotor task on a trial-by-trial basis. We found a significant decrease in activity in the right middle frontal gyrus and bilateral inferior parietal lobules along the task (P < 0.05, FDR corrected).

### Correlation analysis

**Figure 4:**
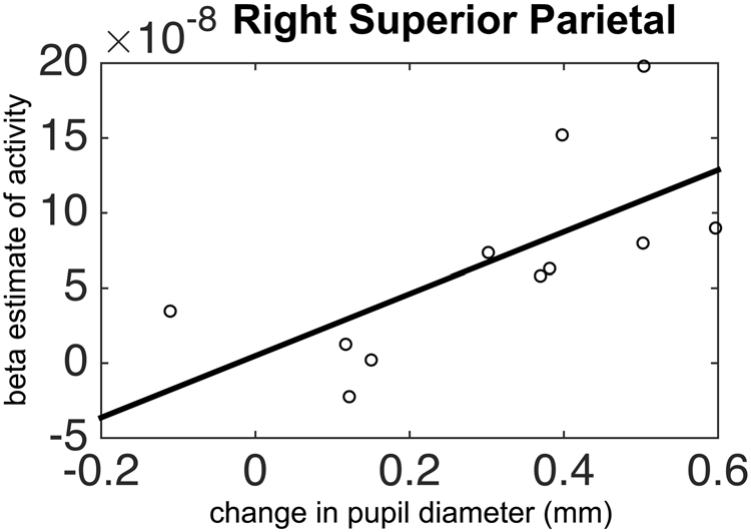
Association between pupillary response and brain activity. A significant positive correlation was observed between average pupil dilation and changes in activity in the right superior parietal region during acceleration trials (P = 0.002).

We also examined the association between behavioral and pupillary responses with cortical activity. We found a significant positive correlation between average pupil dilation and activity in the right superior parietal region during acceleration trials in the first session (R = 0.85, P = 0.002, FDR corrected) (Fig. 4).

## Discussion

We tested the behavioral, pupillary and neural correlates of visuomotor control under different cognitive demands – incongruent steering and unexpected acceleration – using direct and indirect measurements of brain activity – fNIRS and pupillary response, respectively. We identified a cortical region in the right superior parietal lobule that responded to both types of visuomotor cognitive load utilized in our task; this region was significantly active in both sessions of the task suggesting the importance of this region in both early and late stages of visuomotor adaptation. Higher activity in this region was also associated with larger pupillary response in the acceleration trials. On the other hand, regions in the bilateral inferior parietal lobule appeared to be primarily involved in handling visuomotor load associated with acceleration. Our results confirm previous reports regarding the crucial role of parietal regions in visuomotor transformation^3, 18, 21, 23^ and extend them by revealing the unique role of the right superior parietal region in accommodating visuomotor mapping under different types of visuomotor load. Our data also extend previous findings connecting changes in pupil diameter to neural activity under different types of visuomotor cognitive load.

Several cortical regions, including the right superior parietal lobule, bilateral inferior parietal lobule, and right middle frontal gyrus, showed greater activity in the visuomotor task relative to the control task. These results are consistent with previous fMRI findings regarding the involvement of these regions in visuomotor control^5, 7, 8^ and suggests that fNIRS is a good candidate for use in more naturalistic settings such as driving simulators or actual driving conditions.

The superior parietal lobule is part of the dorsal visual pathway and plays an important role in visuospatial and visuomotor processing^24^. Previous fMRI studies suggest the involvement of the superior parietal lobule in visuomotor coordinate transformation^4^ and modulation of visuomotor feedback control^22^. We found that activity in this region was significantly higher in incongruent steering and acceleration trials compared with congruent steering and non-acceleration, suggesting an important role for the superior parietal lobule in accommodating different types of visuomotor cognitive load. Intriguingly, the activity in this region also showed a significant interaction between incongruent steering and acceleration – with higher response to acceleration trials when combined with incongruent steering – making the activity of this region an ideal candidate for objective measurement of visuomotor cognitive loads. Further, our data revealed that the activity in the right superior parietal lobule was sustained across both sessions. A number of studies suggest that superior parietal regions are involved in remapping visuomotor coordinates and thus mainly contribute to early stages of adaptation^6, 18^. Companion studies have indicated the involvement of this region in storing the learned visuomotor map and supports its involvement in late stages of visuomotor learning^17, 20, 21^. Other studies reported stable activity in this region throughout the adaptation process^9, 22^. Our data corroborate the latter findings and suggest that the activity in the right superior parietal region may reflect visuomotor cognitive load and thus signal the importance of this region in both early and late stages of visuomotor adaptation.

The inferior parietal lobule was the other active region during visuomotor performance. The results suggest that activity in this region was significantly higher in the acceleration trials compared with no acceleration across both sessions. Previous data associated activity in the inferior parietal lobule with retrieving predictive motor commands^22^, visuomotor monitoring^25^, as well as with visuospatial working memory processes underlying the task^8^. The inferior parietal lobule is part of the frontoparietal working memory network and we speculate that activity in this region is associated with visuospatial working memory demands of the task^8^, since this region was mainly engaged in visuomotor performance in the first session. This idea is further supported by the observation that indicated a significant decrease in activity of this region along task trials.

The right dorsolateral prefrontal cortex also showed significantly greater activity in the visuomotor task compared with the control condition. Previous fMRI studies on visuomotor control have associated activity in this region with visuospatial working memory processing^8^, acquisition or encoding of explicit rules^9^, and monitoring and detection of visuomotor incongruence^25^. The involvement of prefrontal regions in visuomotor control has also been shown in previous fNIRS studies^26^. However, we did not find significant increase in activity of this region during incongruent or acceleration trials. Previous studies have shown that visuomotor tracking skills are not impaired in patients with dorsolateral prefrontal damage^27^, in contrast to patients with parietal damage^21^. Thus, our data favor an explicit role for parietal regions in handling visuomotor cognitive load compared with the prefrontal cortex. The activity in this region also showed significant adaptation to the task along trials, further supporting the involvement of this region in visuospatial working memory processes associated with the task. It should be noted that we could not interrogate regions in the medial prefrontal and anterior cingulate cortices that play a crucial role in tracking incongruency and conflict monitoring because of limited spatial coverage of fNIRS. Future fMRI studies can further investigate the distinct role of the frontal – parietal network in response to various types of visuomotor cognitive load.

We also continuously measured pupillary response during visuomotor performance. Our pupillometry data showed a significant increase in pupil size in the incongruent and acceleration trials. Behavioral studies have consistently linked pupillary dilation to attentional load in a variety of tasks including visual target detection^28^, number processing and working memory^15^, visuospatial judgment^29^, and multisensory integration^30^. Our data support these findings and signal the higher attentional effort demanded in incongruent steering and acceleration conditions. Further, change in pupil size was significantly associated with the activity in the right superior parietal region. Pupillary response has been linked to activity in the LC and noradrenergic system in animal and human studies^13, 16^. The LC connections are widely distributed across the brain but regions involved in visuospatial attention, particularly the regions in the parietal cortex, receive dense LC inputs^31^. The strong association between activity in the right superior parietal lobule and pupillary dilation in our study further confirms that activity in this region arises from the need for increased attentional effort and alertness for visuomotor control in the acceleration trials. This idea is further corroborated by a recent study that showed a link between pupillary dilation and activity in the superior parietal lobule during a multi-object tracking task^13^. Our data suggest that pupillometry data can supplement the fNIRS measurements and are quite beneficial in interpretation of these data.

Together, our data shed light on cortical regions that accommodate visuomotor mapping under different cognitive demands. Particularly, the results suggest the unique role of the right superior parietal lobule in handling different types of visuomotor cognitive load in early and late stages of visuomotor adaptation. In addition, concurrent measurement of behavior, pupil dilation and fNIRS cortical activity allowed us to more accurately describe the human response to visuomotor cognitive load. Further, employing wearable fNIRS and eye tracking systems enable investigators interested in this and similar brain-behavior associations to examine cognitive load in more naturalistic situations. In particular, the results presented here have important implications for monitoring of cognitive load in real-world tasks such as driving and sports.

## Methods

### Participants

Twenty-three healthy adults (12 females, mean age: 25.02 years) were recruited for participation in the study (N_fNIRS_ = 20, N_eye-tracking_ = 11). Participants were excluded if they reported any clinical psychiatric history such as anxiety or depression, or other chronic or significant medical conditions. The participants were recruited via mailing lists and ads/flyers. Written informed consent was obtained from the participants prior to participation. The Stanford University Institutional Review Board approved the study. The experiment was performed in accordance with relevant guidelines and regulations.

### Task paradigm

We designed a computer-based visuomotor game, in which the participant was instructed to navigate an object, denoted as a “car”, down the center of a winding road (Fig. 1). The car objects, which varied across experimental conditions (i.e., task vs. control), were represented by a circle or square. In the task condition, the car was a circle and the participant was instructed to steer the car with the left and right arrow keys on a standard QWERTY computer keyboard. We manipulated the difficulty of the task trials by reversing the mapping between visual and motor response (i.e., congruent: right/left key = right/left direction; incongruent: right/left key = left/right direction) and also by unexpectedly increasing the speed of the car. The steering congruence was held constant across each trial. On trials in which the speed of the car was increased, the point at which the speed increase occurred was random, but persisted throughout the duration of the trial. The participants were instructed to stay as close to the center of the road as possible at all times. If the car was steered off the road, the participant was instructed to return to the middle of the road as quickly as possible.

In order to control for neural processes associated with visually tracking the car, we designed a control condition in which the car object was a square in the beginning of the trial but changed between a square and a circle multiple times throughout the trial. The participants were instructed that the car would drive autonomously on control trials, so that steering was not required. Instead, the participant was instructed to press any button each time the car changed shape from a square to a circle. The same proportion of trials containing a speed increase within the task condition was also used within the control condition.

The task consisted of a total of 108 trials broken evenly into two separate sessions (~20 min each) with a break in between. Each of the three steering conditions (congruent steering, incongruent steering and control) was repeated 36 times. For each of the three steering conditions, half of the trials included unexpected acceleration. The order of the trials was pseudorandom with a jittered fixation (7 s–9 s). Three different types of roads were used (Fig. 1A) and the composition of road types was similar across conditions. The trials were self-paced and the duration of a trial within condition was dependent on subject’s performance, allowing subjects to complete the navigation at their own pace.

Stimuli were presented on a 20″ LED monitor. Task responses were collected by button presses using right-hand index and middle fingers on a standard keyboard. Participants were seated 70 cm from the screen. Each participant completed five practice trials to become familiar with the task. We quantified subjects’ performance in each trial as the mean distance of the car from the center of the road over the duration of the trial.

### fNIRS and eye tracking data acquisition

A Tandem NIRSport (NIRx, Germany) system was used for fNIRS measurements recording at 7.81 Hz and employing two different wavelengths (760 nm & 850 nm) to measure relative changes of oxygenated and deoxygenated hemoglobin. Thirty-two optodes consisted of 16 LED illumination time-multiplexed sources and 16 detection sensors were used for data acquisition. Optodes were evenly distributed among 4 different regions of interest consisting of 10 channels each; left prefrontal, right prefrontal, left parietal and right parietal cortices. Optodes were secured onto participant’s heads by individually sized caps designed for neuroimaging applications (Brain Products, Germany). The optode locations were cut into each cap at size-specific 10/20 locations. Plastic supports placed between each source/detector pair that constituted a recording channel maintained a 3 cm channel length for all participants. In this manner, the arrays were consistently placed on specific 10/20 locations of interest despite changes in head size across participants^32, 33^. This consistency allowed us to choose the fNIRS channels of interest that directly measure each region of interest (see Functional Localization section below). We could not collect fNIRS data for three participants either because of technical issues (one participant) or because of poor signal quality for participants with long, dark and bulky hair (two participants). Complete NIRS data were collected for twenty participants.

Following the fNIRS cap setup and calibration, the eye tracking glasses were set and calibrated to the participant’s head. A SMI ETG 2.0 (SensoMotoric Instruments, Germany) was used for eye tracking measurements. The eye tracking glasses were binocular, allowing recording of pupillary data by two infrared cameras (one for each eye) integrated in the inner eyeglass frame. The glasses recorded at a sampling rate of 30 Hz. Pupillary data were collected only from a subset of participants (N = 11).

### Functional Localization

A region of interest (ROI) was defined by grouping channels together with the same source illuminator; 16 total source illuminators generated 16 total ROIs. In order to reduce the overall spatial coverage included in our analysis of cortical activation, we employed a functional localization procedure for each participant that identified the single channel within each ROI that responded greatest to each condition. Functional localization was accomplished by selecting the largest mean beta contrast for conditions across each channel within each ROI. The localized channel within each region of interest was then submitted for statistical analysis.

### Statistical analysis

#### Behavioral data analysis

We quantified subject’s performance in each trial as the mean distance of the car from the center of the road over the duration of the trial. The mean performance across conditions was then submitted to a three-way repeated measures ANOVA with steering (congruent and incongruent), acceleration (no acceleration, with acceleration) and road difficulty (easy, medium, difficult) as three factors. This analysis was also performed separately for each session to examine changes in performance in early and late stages of the visuomotor task. The deviation from the center was zero in control trials as the car would drive autonomously along the center of the road.

#### fNIRS data analysis

fNIRS data quality checking was carried out using our in-house package^34^ and fNIRS preprocessing was performed according to the pipeline described by Brigadoi and colleagues (2014) using Homer 2 package in Matlab^35, 36^. Specifically, optical density data were corrected for motion artifacts using wavelet motion correction procedure. Then, a band-pass filter with cutoff frequency of 0.01 Hz and 0.5 Hz was applied^34^ before converting the optical density data to oxygenated and deoxygenated hemoglobin using the modified Beers-Lambert law^37^. The deoxygenated hemoglobin was used as a proxy of brain activity as it previously showed maximum agreement with functional MRI BOLD signal (R = 0.98)^38–40^. After preprocessing, a general linear model was first performed at the individual level.

The task design was modeled as a boxcar, convolved with a hemodynamic response. The model fit was performed for each individual subject across all channels and beta estimates of activity associated with each of the six conditions were estimated. We restricted the analysis to cortical regions that showed greater activity in all task conditions compared with control. To this purpose, the beta estimates for all task conditions were contrasted against corresponding control and the contrasted beta was submitted to a one-sample t-test (p < 0.05, FDR corrected).

To identify the regions that differentially respond to cognitive load associated with incongruent steering, increasing speed and road difficulty, a three-way repeated measures ANOVA was conducted on beta estimates of activity with steering (congruent and incongruent), acceleration (no acceleration, with acceleration) and road difficulty (easy, medium, difficult) as three factors of interest. This analysis was also performed separately for each session to distinguish the brain regions adapting to novel visuomotor mapping and increased speed in early and late stages of the visuomotor task. Further, to examine the cortical regions that adapt to visuomotor task on a trial-by-trial basis, we ran a separate model with trial number as a parametric modulator.

#### Eye tracking data analysis

Pupillometry data were preprocessed using the procedure described in ref. 41. Artifacts, including blinks, were identified and replaced using linear interpolations. Data were smoothed using a five-point unweighted average and were detrended to eliminate the slow drift in pupil diameter. The average changes in left and right pupil diameter in the task trials relative to control were quantified and submitted for statistical analysis. To examine the differences in pupil dilation in response to cognitive load associated with incongruent steering, increasing speed and road difficulty, a three-way repeated measures ANOVA was performed on average change in pupil diameter with steering (congruent and incongruent), acceleration (no acceleration, with acceleration) and road difficulty (easy, medium, difficult) as three factors. Similar to NIRS analysis, this analysis was also performed separately for each session to examine changes in pupil dilation in early and late stages of the visuomotor task.

Finally, we performed Spearman’s correlation analysis to quantify the potential association between behavioral and pupillary responses with cortical activation.

## References

[1] Vaillancourt DE (2003). Neural Basis for the Processes That Underlie Visually Guided and Internally Guided Force Control in Humans. Journal of Neurophysiology.

[2] Culham JC, Cavina-Pratesi C, Singhal A (2006). The role of parietal cortex in visuomotor control: what have we learned from neuroimaging?. Neuropsychologia.

[3] Gottlieb J (2007). From Thought to Action: The Parietal Cortex as a Bridge between Perception, Action, and Cognition. Neuron.

[4] Grefkes C, Ritzl A, Zilles K, Fink GR (2004). Human medial intraparietal cortex subserves visuomotor coordinate transformation. NeuroImage.

[5] Murray EA, Bussey TJ, Wise SP (2000). Role of prefrontal cortex in a network for arbitrary visuomotor mapping. Exp Brain Res.

[6] Graydon FX, Friston KJ, Thomas CG, Brooks VB, Menon RS (2005). Learning-related fMRI activation associated with a rotational visuo-motor transformation. Cognitive Brain Research.

[7] Staines WR, Padilla M, Knight RT (2002). Frontal-parietal event-related potential changes associated with practising a novel visuomotor task. Brain Res Cogn Brain Res.

[8] Anguera JA, Reuter-Lorenz PA, Willingham DT, Seidler RD (2010). Contributions of Spatial Working Memory to Visuomotor Learning. Journal of Cognitive Neuroscience.

[9] Floyer-Lea A (2004). Changing Brain Networks for Visuomotor Control With Increased Movement Automaticity. Journal of Neurophysiology.

[10] Hikosaka O, Nakamura K, Sakai K, Nakahara H (2002). Central mechanisms of motor skill learning. Current Opinion in Neurobiology.

[11] Bunge SA, Hazeltine E, Scanlon MD, Rosen AC, Gabrieli JDE (2002). Dissociable Contributions of Prefrontal and Parietal Cortices to Response Selection. NeuroImage.

[12] Unsworth N, Robison MK (2016). Pupillary correlates of lapses of sustained attention. Cogn Affect Behav Neurosci.

[13] Alnaes D (2014). Pupil size signals mental effort deployed during multiple object tracking and predicts brain activity in the dorsal attention network and the locus coeruleus. Journal of Vision.

[14] Wierda SM, van Rijn H, Taatgen NA (2012). Pupil dilation deconvolution reveals the dynamics of attention at high temporal resolution. Proceedings of National Academy of Sciences.

[15] Kang OE, Huffer KE, Wheatley TP (2014). Pupil dilation dynamics track attention to high-level information. PLoS ONE.

[16] Murphy PR, O’Connell RG, O’Sullivan M, Robertson IH, Balsters JH (2014). Pupil diameter covaries with BOLD activity in human locus coeruleus. Hum. Brain Mapp..

[17] Sakai K (1998). Transition of brain activation from frontal to parietal areas in visuomotor sequence learning. J. Neurosci..

[18] Inoue K (1997). Activity in the parietal area during visuomotor learning with optical rotation. Neuroreport.

[19] Kawashima R, Roland PE, O’Sullivan BT (1995). Functional Anatomy of Reaching and Visuomotor Learning: A Positron Emission Tomography Study. Cerebral Cortex.

[20] Haar S, Donchin O, Dinstein I (2015). Dissociating visual and motor directional selectivity using visuomotor adaptation. Journal of Neuroscience.

[21] Cavaco S, Anderson SW, Chen K-H, Teixeira-Pinto A, Damasio H (2015). Parietal damage impairs learning of a visuomotor tracking skill. Neuropsychologia.

[22] Grafton ST, Schmitt P, Van Horn J, Diedrichsen J (2008). Neural substrates of visuomotor learning based on improved feedback control and prediction. NeuroImage.

[23] Buneo CA, Andersen RA (2006). The posterior parietal cortex: Sensorimotor interface for the planning and online control of visually guided movements. Neuropsychologia.

[24] Halsband U, Lange RK (2006). Motor learning in man: A review of functional and clinical studies. Journal of Physiology-Paris.

[25] Schnell K (2007). An fMRI approach to particularize the frontoparietal network for visuomotor action monitoring: Detection of incongruence between test subjects’ actions and resulting perceptions. NeuroImage.

[26] Gentili RJ, Shewokis PA, Ayaz H, Contreras-Vidal JL (2013). Functional near-infrared spectroscopy-based correlates of prefrontal cortical dynamics during a cognitive-motor executive adaptation task. Front. Hum. Neurosci..

[27] Gomez Beldarrain M, Gafman J, Ruiz de Velasco I, Pascual-Leone A, Garcia-Monco J (2002). Prefrontal lesions impair the implicit and explicit learning of sequences on visuomotor tasks. Exp Brain Res.

[28] Privitera CM, Renninger LW, Carney T, Klein S, Aguilar M (2010). Pupil dilation during visual target detection. Journal of Vision.

[29] Verney SP, Granholm E, Dionisio DP (2001). Pupillary responses and processing resources on the visual backward masking task. Psychophysiology.

[30] Rigato S, Rieger G, Romei V (2016). Multisensory signalling enhances pupil dilation. Sci. Rep..

[31] Benarroch, E. E. The locus ceruleus norepinephrine system. *Neurology* (2009). 10.1212/WNL.0b013e3181c2937c 19917994

[32] Okamoto M (2004). Three-dimensional probabilistic anatomical cranio-cerebral correlation via the international 10–20 system oriented for transcranial functional brain mapping. NeuroImage.

[33] Tsuzuki D (2012). Stable and convenient spatial registration of stand-alone NIRS data through anchor-based probabilistic registration. Neuroscience Research.

[34] Cui X, Bray S, Reiss AL (2010). Functional near infrared spectroscopy (NIRS) signal improvement based on negative correlation between oxygenated and deoxygenated hemoglobin dynamics. NeuroImage.

[35] Brigadoi S (2014). Motion artifacts in functional near-infrared spectroscopy: A comparison of motion correction techniques applied to real cognitive data. NeuroImage.

[36] Huppert TJ, Diamond SG, Franceschini MA, Boas DA (2009). HomER: a review of time-series analysis methods for near-infrared spectroscopy of the brain. Appl Opt.

[37] Wyatt JS, Delpy DT, Cope M, Wray S, Reynolds EOR (1986). Quantification of cerebral oxygenation and haemodynamics in sick newborn infants by near infrared spectrophotometry. The Lancet.

[38] Huppert TJ, Hoge RD, Diamond SG, Franceschini MA, Boas DA (2006). A temporal comparison of BOLD, ASL, and NIRS hemodynamic responses to motor stimuli in adult humans. NeuroImage.

[39] Huppert, T. J., Hoge, R. D., Franceschini, M. A. & Boas, D. A. A spatial-temporal comparison of fMRI and NIRS hemodynamic responses to motor stimuli in adult humans. in (eds Chance, B., Alfano, R. R., Tromberg, B. J., Tamura, M. & Sevick-Muraca, E. M.) **5693**, 191 (SPIE, 2005).

[40] Wijeakumar S, Huppert TJ, Magnotta VA, Buss AT, Spencer JP (2016). Validating an image-based fNIRS approach with fMRI and a working memory task. NeuroImage.

[41] Siegle GJ, Steinhauer SR, Stenger VA, Konecky R, Carter CS (2003). Use of concurrent pupil dilation assessment to inform interpretation and analysis of fMRI data. NeuroImage.

